# The Possibility of Deep Learning-Based, Computer-Aided Skin Tumor Classifiers

**DOI:** 10.3389/fmed.2019.00191

**Published:** 2019-08-27

**Authors:** Yasuhiro Fujisawa, Sae Inoue, Yoshiyuki Nakamura

**Affiliations:** Department of Dermatology, University of Tsukuba, Tsukuba, Japan

**Keywords:** artificial intelligence, deep learning, convolutional neural network, clinical image, dermoscopy, skin tumor classifier

## Abstract

The incidence of skin tumors has steadily increased. Although most are benign and do not affect survival, some of the more malignant skin tumors present a lethal threat if a delay in diagnosis permits them to become advanced. Ideally, an inspection by an expert dermatologist would accurately detect malignant skin tumors in the early stage; however, it is not practical for every single patient to receive intensive screening by dermatologists. To overcome this issue, many studies are ongoing to develop dermatologist-level, computer-aided diagnostics. Whereas, many systems that can classify dermoscopic images at this dermatologist-equivalent level have been reported, a much fewer number of systems that can classify conventional clinical images have been reported thus far. Recently, the introduction of deep-learning technology, a method that automatically extracts a set of representative features for further classification has dramatically improved classification efficacy. This new technology has the potential to improve the computer classification accuracy of conventional clinical images to the level of skilled dermatologists. In this review, this new technology and present development of computer-aided skin tumor classifiers will be summarized.

## Introduction

The incidence of skin cancers, including melanoma and non-melanoma skin cancers (NMSC), is globally increasing. In the United States, the incidence of melanoma is reported to be 22.1 per 100,000 people, the number of new yearly melanoma patients is estimated to be more than 63,000, and melanoma is now rated as the 6th most common of all cancers (1). In spite of new therapeutic agents, such as checkpoint and BRAF inhibitors which improve survival of advanced cases, melanomas are still lethal (2–4). On the other hand, most NMSCs, which are responsible for 4.3–5.4 million new cases each year in the United States (5, 6), can be treated simply by surgical removal. Most of these (>90%) are comprised of basal cell carcinomas (BCC) and squamous cell carcinomas (SCC) (7) and skilled dermatologists can detect these by clinical appearance and a tumor magnifying dermatoscope (8). Consequently, most SCCs and BCCs are detected at an early stage and resolved by surgery alone. However, SCC can become lethal when it metastasizes, since few standardized and effective therapies for advanced SCC have been established. Although metastatic BCC is very rare, any delay in diagnosis may allow tumors to become unresectable. Therefore, early detection of all skin cancers, not limited to melanoma, is required to prevent progression of these cancers to advanced stages and reduce skin cancer-related deaths.

Skin tumor screening is one solution for the early detection of skin cancer, but it is not practical for dermatologists to check all patients for skin tumors. In most countries, primary vigilance is maintained through primary care clinics before being referred to dermatologists and, consequently, up to 20% of patients consult at primary care clinics complaining about skin-related symptoms (9–11). A study by Julian et al. reported that 16% of patients with dermatologically related diseases had benign skin tumors, 3.3% had actinic keratosis, and 3% had malignant skin tumors (10), meaning ~20% of patients who consulted a primary care doctor with skin-related complaints received a tumor diagnosis. Similar results were reported by Kerr et al. (11), showing that 11.4% of studied patients had benign skin tumors with <5% having malignant skin tumors. Although the percentage of patients with malignant skin tumors among patients who consult at primary care clinics is not so high, primary care doctors are under a heavy burden to correctly screen patients who present with skin-related symptoms and determine which patients are to be transferred to the dermatologists. Thus, any device or service that can accurately give the probability of malignancy by analyzing a simple photograph of the tumor would be very helpful for both primary care doctors and their patients. In this context, the development of artificial intelligence (AI) that can classify skin tumor images within seconds, at a skill level similar to trained dermatologists, is an ideal solution for this problem.

## Machine Learning: Necessity of Labeled Data

**Figure 1:**
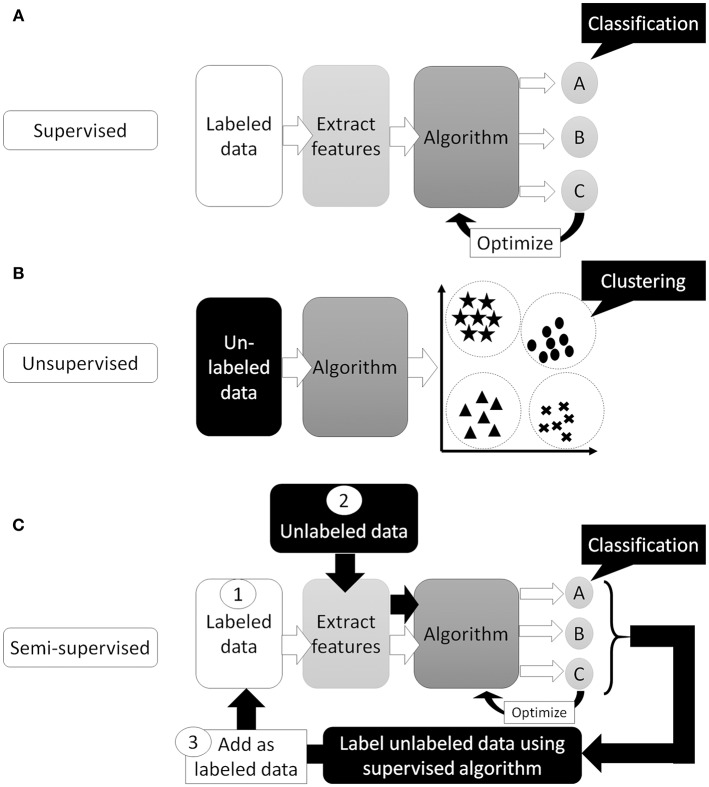
Supervised or unsupervised training. **(A)** Supervised training which can predict classification or regression of the input data. **(B)** Unsupervised training which can cluster the data. **(C)** Semi-supervised training. First, train the algorithm by small number of labeled data. Then, use trained algorithm to “label” unlabeled data. Next, re-train algorithm using newly labeled data and originally labeled data. Finally, algorithm trained with all data and can predict classification or regression of the input data.

**Figure 2:**
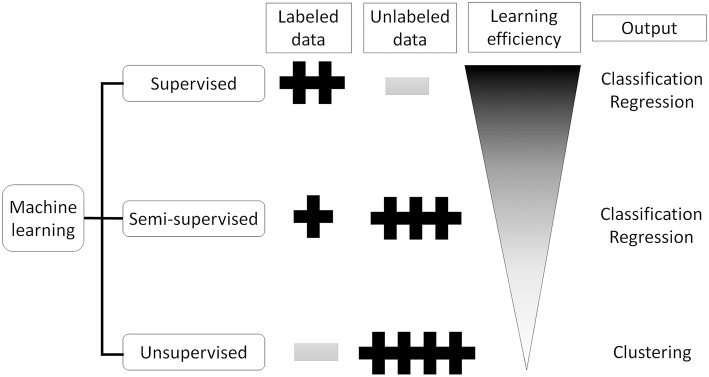
Supervised, semi-supervised, and unsupervised training. Supervised training needs labeled data but can learn the most efficient. Unsupervised training does not need labeled data which sometimes difficult to prepare, but can only cluster the input data. Semi-supervised training can produce labeled data from unlabeled data using small number of labeled data.

Artificial Intelligence (AI) is a term used to describe machine software that can mimic human cognitive functions, such as learning and problem solving (12). Machine learning achieves this via changes in the program algorithm that allow it to complete tasks more efficiently. These changes come from training using labeled data (supervised learning method, Figure 1A), data without labels (unsupervised learning method, Figure 1B), or both (semi-supervised learning method, Figure 1C) (13). For the supervised learning method, the program processes data and compares its output with the correct answer (label), adjusting its own parameters so that it can reach the correct result. This process should be repeated for as many training datasets as are available but, to achieve satisfactory efficiency, it requires a certain amount of labeled data to adjust the parameters. Thus, preparing a high enough number of datasets means that this supervised learning method could achieve a high classification efficacy (Figure 2). However, preparation of labeled data is often difficult, especially in the medical field. On the other hand, unsupervised learning does not require labeled data but instead uses a large amount of unlabeled data for learning. Where it differs from the supervised method is in the output of this algorithm, which clusters data instead of classifying it (Figure 1B). This method is useful for huge amounts of data without labels but, since this algorithm does not know the “answer,” the meaning of each cluster in the output needs to be determined. The third method, semi-supervised learning, requires a small amount of labeled data with a large amount of unlabeled data. It functions on the principle that unlabeled data is classified using an algorithm trained with labeled data (Figure 1C). These unlabeled data are labeled with categories that were found to have high hit probability as calculated by the algorithm which was trained (supervised learning method) on the small amount of labeled data. These newly labeled data are added to the originally labeled data and supervised learning is then conducted again to re-train the algorithm. This method is useful for huge amounts of unlabeled data that would otherwise require a high cost to label. However, if a small error exists in the initial supervised learning algorithm, that error will be amplified at the end of the procedure. Collectively, the best way to train AI algorithms is to prepare large amounts of correctly labeled data for supervised learning but this is often difficult for the medical field, since the “gold standard” pathological confirmation of lesion labels requires the excision of “all” lesions, which is not ethical for confirmed, benign lesions.

## Method of Machine Learning for Image Classification: Before the Deep Learning Era

**Figure 3:**
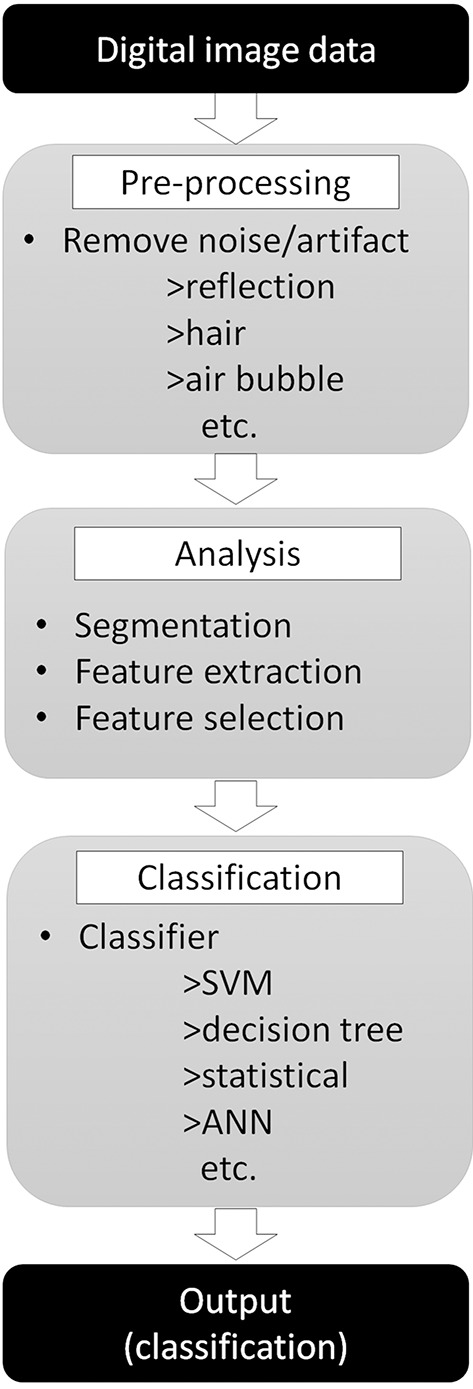
Skin tumor classifier by “traditional” machine learning. Digital image data needs pre-processing to remove noise or artifact to improve the efficacy of the next step. Pre-processed images then analyzed to extract features required for classification step. Finally, classifier use extracted features to classify input images.

Developing a computer-aided diagnostic support system for skin cancer diagnosis requires many steps, as reviewed by Masood and Al-Jumaily (14) (Figure 3). The first step of the classification process starts by removing irrelevant structures and artifacts in the image (14, 15), such as hair, air bubbles/gel (if dermoscopy images are used), ink markings or reflectance, by using general image filters (16). These irrelevant objects or artifacts may affect the efficacy of border detection and skew the final output. Next, to analyze the internal properties for further analysis, the lesion within the objective tumor area should be separated from the surrounding skin in a procedure called segmentation. As it is not practical to manually define areas for all images, many automatic lesion segmentation systems have been reported (17–19), but this step is still a challenging task for engineers (16).

Next, the important features need to be extracted from the segmented image. Border shapes [asymmetry indices, symmetry axes, or aspect ratios (20)] or color features [average values and standard deviation of the RGB or HSV color channels (21)] are calculated and these values are used for further classification. However, there is a cost for attempting to extract more features, namely more training time, more complex algorithms, less generalization behavior, and less prediction accuracy. Thus, it is important to select only the useful feature values for classification while eliminating less useful ones (feature selection). There are diverse and numerous methods that have been proposed for this feature selection process (22–24).

**Figure 4:**
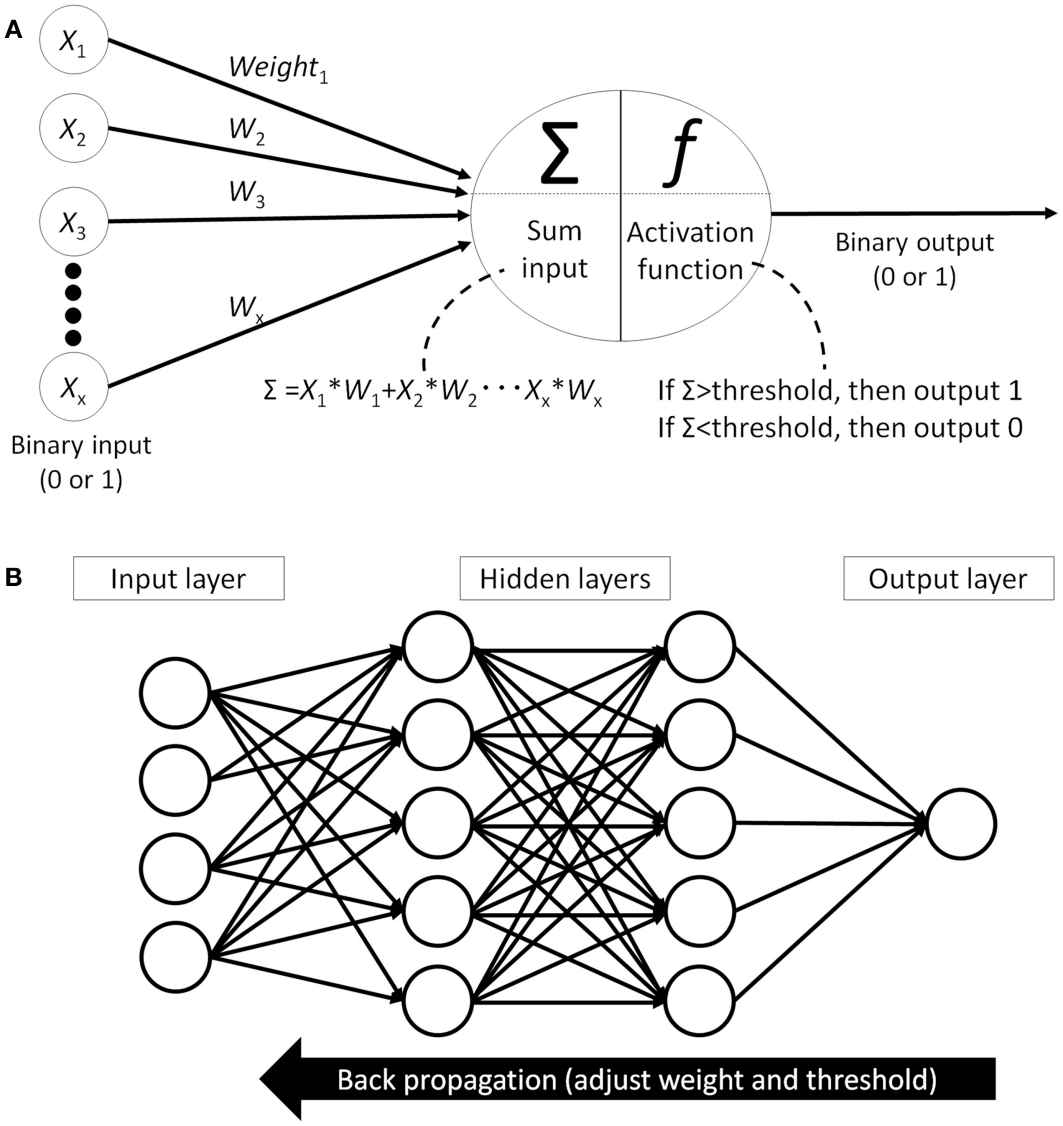
Artificial neural network (ANN). **(A)** Single perceptron model which mimics the structure of biological neural networks in the human brain. Each node receives signal from other nodes (X1, X2… Xx). Add the multiplied values of input and weight (W) and when this sum(Σ) cross the threshold, then this node outputs signal. **(B)** An example of artificial neural network model which has hidden layer between input layer and output layer. All the nodes between the layers are fully connected and each connection has weight. Machine learning is adjusting each weights and thresholds in the network to reach the correct output (back propagation).

Finally, the classification algorithm outputs the result using the selected feature values calculated in the previous phase. There are many different algorithms available for this classification task: support vector machine (25), decision tree (26), statistical [logistic regression (27)], or artificial neural network (ANN) (28). Of those, the performance of the support vector machine classifier is reported to be similar or better than other algorithms but, as it can only provide a dichotomous distinction between two classes (e.g., benign or malignant), this algorithm will not work for multi-class sorting with probabilities for each class. ANN, on the other hand, mimics the structure of biological neural networks in the human brain (Figure 4A) and can change connectivity between decision nodes (back propagation, Figure 4B) so that the network can achieve satisfactory results (28). Many ANN studies have reported on dermoscopic image analysis as (28) it has the ability to derive meaning from data which is too complex for humans to understand. The downside, however, is that ANN requires multiple repetitions of the training data to adjust network connections.

## Introduction of Deep Learning Technology with Convolutional Neural Networks

The image classification machine learning algorithms described above are very complex; they are based on hand-engineered features and are highly dependent on prior knowledge. For example, in some reports, more than 50 different feature values thought to be useful in the classification process, such as color, shape, or border information, were extracted from a single image for the training of the system (29, 30). In the annual ImageNet Large Scale Visual Recognition Challenge (ILSVR) computer vision competition, where 1 to 2 million images of objects are classified into 1,000 categories, a classifier using traditional machine learning had an error rate of 30% (31) compared to humans who logged an error rate of 5.1% (32). This striking gap in accuracy was dramatically reduced in the 2012 ILSVR competition; deep-learning technology using a convolutional neural network (CNN) achieved an error rate of 16.4% while other classifiers using traditional machine learning had an error rate of 26–30% (32). After the introduction of CNN, the error rates in the ILSVR competition dropped rapidly and the error rate in the 2017 competition was below 5%, indicating that CNN classified images more precisely than humans (http://image-net.org/challenges/LSVRC/2017/results).

**Figure 5:**
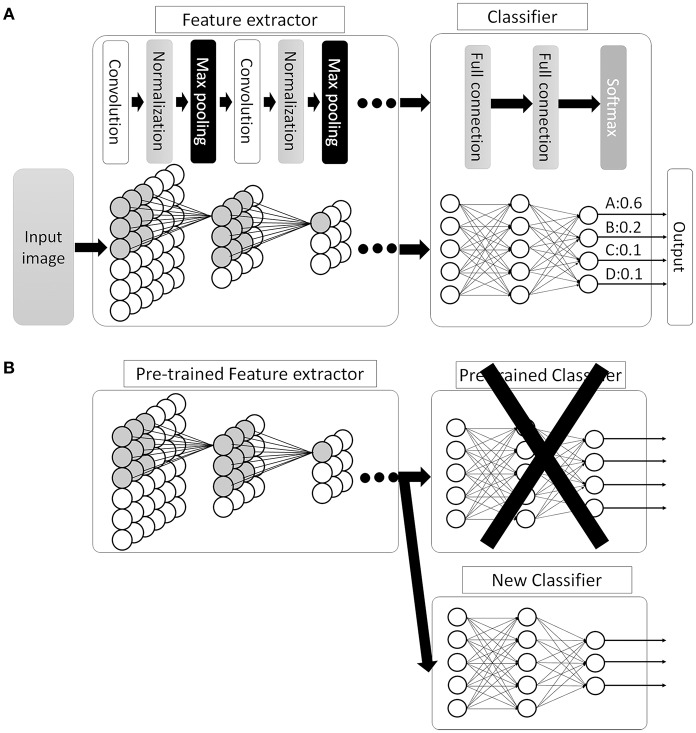
Convolutional neural network (CNN). **(A)** Schematic image of CNN. Between the convolutional layers, each nodes connect to distinct nodes of the previous layer, which is different compared with ANN (as in Figure 4B, all the nodes between the layers are fully connected). By this feature, CNN can successfully capture the spatial and temporal dependencies in an image. Then, the fully connected classifier output the result as a probability distribution. **(B)** An example of transfer learning in CNN. In this example, replace classifier and use pre-trained CNN layers as feature extractor. Then, train the system to fit the new task.

This new CNN technology can learn and automatically determine what features are important for classification from the training image set. The extraction and selection of the features for classification was a key component of the traditional methods (33), and also the most difficult part. Thus, by using CNN, complicated image pre-processing is no longer necessary to obtain optimal feature values for the image classification. A schematic structure of CNN is shown in Figure 5A. In ANN, every node fully connects to the next layer (Figure 4B) but, in CNN, each node connects only to some nodes in the next layer (Figure 5A). This key feature of CNN can successfully capture the spatial and temporal dependencies in an image through the application of relevant filters (34). In this type of classifier, output values of the feature extractor usually input to a fully connected network and the softmax function finally converts input vectors to real numbers for normalization into a probability distribution (35). As an example, if the input images had 4 different classes, the final CNN layer would have 4 nodes as in Figure 5A. But, as the sum of the output of all 4 nodes would not be 1, it would be difficult to interpret the output. However, a softmax function that converts each node's output from 0 to 1 would allow for the components to add up to 1 and result in a final output that can be interpreted as a probability (0–100% probability).

There are many available CNN architectures used in the medical field such as LeNet (36), AlexNet (36), ZFNet (37), VGGNet (38), GoogLeNet (39, 40), ResNet (41), or SENet (42) (Table 1). Not only are these architectures free to use, pre-trained models are also available that are commonly trained by the previously mentioned ILSVR2012 dataset, which contains 1.2 million images within 1,000 classes (available at http://image-net.org/download-imageurls). Since the ability to extract image features by pre-trained models is very high, we can use these pre-trained models as a “feature extractor” in a technique called transfer learning (43). One example of transfer learning is shown in Figure 5B. Basically, the classifier part is replaced with an untrained classifier appropriate to the new task and the system is trained using a new training image dataset. This method is useful when large numbers of datasets cannot be prepared due to rarity, expense in collection/labeling, or inaccessibility (43). Therefore, transfer learning would be useful for the medical field since it is often difficult to collect images of rare diseases.

**Table 1:** List of CNN architectures.

**Architecture**	**Year**	Top-5 error rate at ILSVRC^*^	**References**
LeNet	1998	NA	(36)
AlexNet	2012	15.3%	(36)
ZFNet	2013	14.8%	(37)
GoogLeNet	2014	6.67%	(39, 40)
VGG Net	2014	7.3%	(38)
ResNet-50	2015	3.6%	(41)
SENet	2017	2.3%	(42)

* *ILSVRC: ImageNet Large Scale Visual Recognition Challenge*.

Collectively, the introduction of CNN has not only dramatically improved image classification efficacy, it has also made adoption of machine learning and image classification easier and cheaper since most of the initially needed resources are easily accessible.

## Skin Tumor Classification by Using Dermoscopic Images

The clinical diagnosis of melanoma is difficult since the morphological characteristics of other pigmented skin lesions may sometimes mimic it. Dermoscopy can magnify the skin and enables clinicians to better evaluate morphological features which are difficult to see with the naked eye. The introduction of dermoscopy has been reported to improve diagnostic sensitivity by 10–30% (44–46). Physicians are usually taught the ABCD-rule (47), Menzie's method (48), 7-point checklist (48), or some other pattern classification methods (49) to distinguish between melanoma and non-melanoma pigmented skin lesions. However, becoming an experienced dermoscopic reader (50, 51) who can score 90% diagnostic sensitivity (proportion of images correctly detected as malignant within all malignant images) and 59% diagnostic specificity (proportion of images correctly detected as benign within all benign images) requires a significant time and training investment (52). Moreover, even after such training, the readings are often complex and subjective.

To make readings more objective and qualitative, as well as support physicians using dermoscopy, many computer-based analyses of dermoscopic images to classify melanomas have been conducted. In a 2009 review by Rajpara et al. (53) that reviewed studies of AI classifiers (12 studies) and dermoscopy (23 studies) published between 1991 and 2002, the melanoma detection sensitivity and specificity of AI classifiers were already similar to that of physicians using dermoscopy; pooled sensitivity, and specificity for AI classifiers and physicians were 91 vs. 88% and 86 vs. 79%, respectively. In a 2013 review (14), 15 new studies on AI classifiers were included and showed sensitivities ranging from 60.7 to 98% and specificities ranging from 72 to 100%, which were similar to the previous 2009 report. Haenssle et al. reported on an AI classifier using the CNN deep learning-based algorithm and compared the classification efficiency against 58 dermatologists (54). Their CNN showed higher accuracy than most dermatologists (sensitivity 88.9 vs. 86.6% and specificity 82.5 vs. 71.3%, respectively) (54). Similar results were recently reported by Tschandl et al. (55) which compared 511 human readers, including 283 board-certified dermatologists, and 139 machine learning algorithms on a classification task consisting of 30 image batches from the test image set. When comparing 37 human experts (>10 years of experience) with the top three machine learning algorithms, the mean number of correctly classified images by humans was 18.78 images per 30 test images, whereas machine learning algorithms scored 25.43, which was statistically higher than human expert readers.

Although AI classifiers seemed to score similar marks as physicians, it is difficult to judge whether AI classifiers have already surpassed physicians or not, since most of these reports were unable to verify results by outside data and biases, such as selection bias of study training/testing data or publication bias (53). However, in spite of such possible biases, development of AI classifiers using dermoscopic images is still an attractive research area.

## Classification of Skin Tumor using Clinical Images

As described above, many highly accurate AI classifiers focusing on melanoma detection using dermoscopic images have been developed. Although conventional machine learning algorithms that require human intervention to extract and select features have the capacity to output a binary result (benign or malignant), accurate diagnoses of multi-class skin diseases are difficult (56). Moreover, even if clinical images are cheap and easy to collect, these clinical photographs are believed to have limited morphologic information that is useful for classification (14). Collectively, outside of a binary “benign or malignant” output, AI classifiers using conventional machine learning algorithms are considered to be inferior at handling clinical images for sorting into multiple classes.

**Figure 6:**
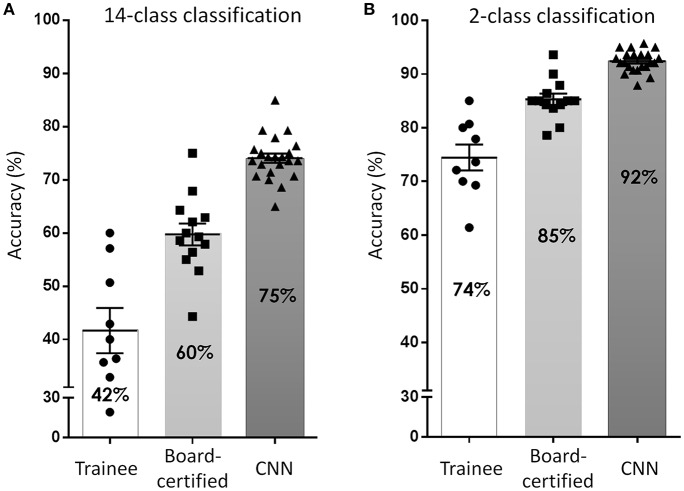
Accuracy of skin tumor classification by our CNN classifier. **(A)** Result of 14-class classification by dermatology trainees, board-certified dermatologists, and CNN classifier. Adapted from Fujisawa et al. (39). **(B)** Result of 2-class classification (benign or malignant). In both classification level, the accuracy of CNN surpassed board-certified dermatologists.

To overcome these issues, deep learning-based CNN classifiers, which surpass the general object classification capability of humans (32), became popular for use in these tasks. Several studies have been published, including from our group (39, 57, 58), since Esteva et al. (40) first reported a dermatologist-equivalent classifier of skin cancers using CNN in 2017. They used 129,450 skin lesion images for the training of a Google Inception v3 CNN architecture, which was pre-trained on the Image Net dataset consisting of 1.28 million images over 1,000 generic object classes. The CNN was fine-tuned to classify skin lesion images by the transfer learning method and was validated on its efficiency of binary classification (benign or malignant). Although the study did not reveal its overall accuracy at skin tumor classification, the CNN surpassed average-level dermatologists in the sensitivity/specificity of classifying epidermal tumors (epidermal cancers and seborrheic keratosis) and melanocytic tumors (melanoma and benign nevi). Han et al. (57) used 15,408 skin lesion images from 12 benign and malignant skin tumors to train a Microsoft ResNet-152 CNN architecture (pre-trained on the same Image Net dataset as above). Similarly, to the Esteva et al. report, they used skin tumor images to fine-tune their CNN which subsequently outperformed 16 dermatologists. They also opened their CNN to the public and it could be externally validated, which was noteworthy. In our study (39), we used only 4,800 skin lesion images from 14 benign and malignant skin tumors to train a GoogLeNet CNN architecture (pre-trained on the same Image Net dataset as above). However, even with less images in the training set, our CNN was more accurate at image classification than 13 board-certified dermatologists and 8 dermatologist trainees (Figure 6), reaching a 96.3% sensitivity, and 89.5% specificity in the detection of skin cancer.

Brinker et al. (58) reported an interesting result showing that a CNN trained only with dermoscopic images could classify clinical melanoma images at a similar level to 145 dermatologists. They trained a Microsoft ResNet-50 CNN architecture (pre-trained on the same Image Net dataset as above) using 2,196 melanomas and 18,566 atypical nevi. This study is particularly interesting because this is the first report to show that dermatologist-equivalent tumor image classification was achieved by a CNN that was not trained by clinical images. This study indicates that CNN may benefit from training with dermoscopic images (that have a higher resolution than clinical images) even for low-resolution classification tasks. Another approach is to combine available data for classification as Yap et al. (59). They used 2,917 cases containing both clinical and dermoscopic images and trained a Microsoft ResNet-50 CNN architecture. They showed that a CNN trained with dermoscopic images had higher accuracy than a CNN trained with clinical images. However, when they trained their CNN on combined feature information from dermoscopic and clinical images, the accuracy outperformed single modal CNN, indicating that both clinical and dermoscopic images have distinct classification information. Collectively, the new machine learning algorithm CNN could be a “breakthrough” for developing a multi-class skin tumor classifier, which can accept clinical tumor images.

## Limitations

Several issues remain for the CNN skin tumor classifier to overcome. First, there are no standardized evaluation test datasets to measure the efficacy of CNN classifiers. However, if the test dataset is known in advance, there is a risk of adapting the CNN classifier to the test dataset. Therefore, it might be better to conduct tests by a third-party organization to measure classification efficiency using closed datasets. Second, datasets used to train the CNN are comprised of regionally homogenous images, e.g., our dataset was composed of nearly 100% Asians. In a study by Han et al. (57), they tested using external tumor images (Edinburgh dataset; available from the Edinburgh Dermofit Image Library) to see if their CNN that was trained on Asian tumor images could also classify tumor images from Caucasian patients. As anticipated, both sensitivity and specificity dropped in this case. Third, although CNN requires an increased number of training datasets to improve classification efficiency, rare tumors and subtypes (such as amelanotic melanoma or pigmented basal cell carcinoma) will always mean a scarcity of available images for these diseases. Fourth, the clinical images were less standardized, with varying camera angles, orientations, multiple skin backgrounds, lighting, and even pen markings or rulers included in the photos (60). According to a study by Narla et al. (60), algorithms are more likely to classify images with rulers as malignant because images with rulers were more likely to be malignant. Fifth, the “black box” nature of CNN makes it impossible to interpret how and why CNN arrived at its output. As an example, Navarrete-Dochent et al. (61) reported that the output of their CNN was affected by the size, rotation, or color tone of images. A similar phenomenon was observed in our system; the output of our CNN was affected by changing the size parameters of the tumor (data not shown). To improve the robustness of the CNN classifier, establishment of an open-access, standardized, large skin tumor image dataset, which includes both rare tumors/subtypes and all ethnicities, is mandatory. Moreover, a robust, standardized measurement method for evaluation and comparison of systems should be established.

## Future Perspective

AI classifiers for the image classification field have been dramatically improved and made more popular by the introduction of CNN. Many strategies, such as creating ensembles of multiple models (62, 63) or using additional information other than image labels, to improve the accuracy of the classifier outside of increasing the number of images for training have been reported (64). Some studies have reported that CNN algorithms have already surpassed the classification efficacy of dermatologists and, in the near future, AI classifiers may gain sufficient sensitivity and specificity to bear the screening burden for detecting malignant skin tumors. Therefore, some physicians may consider AI as a potential threat, but we believe it to be no more than a diagnostic assistance system due to many limitations detailed in previous studies and difficulty in performance comparisons within published results. Besides such limitations, AI classifiers still have important role in assisting non-dermatologist physicians, since most skin cancer patients will consult them before being transferred to dermatologists. Early detection and treatment are both still essential in the management of melanoma and, therefore, an efficient AI classifier would help to “detect” patients in the early stage of the disease. Further research is thus required to both improve classification efficacy and develop independent evaluation methodologies to accurately measure system efficacy. Moreover, integration of other medical information such as vital signs, routine blood testing, or even omics data, may give us new insights into the biology or pathology of the disease.

## Author Contributions

YF, SI, and YN contributed to the conception and design of the study. SI and YF wrote the first draft of the manuscript. All authors contributed to the revision of the manuscript, and read and approved the submitted version.

### Conflict of Interest Statement

The authors declare that the research was conducted in the absence of any commercial or financial relationships that could be construed as a potential conflict of interest.
